# Circulating Magnesium and Risk of Major Adverse Cardiac Events among Patients with Atrial Fibrillation in the ARIC Cohort

**DOI:** 10.3390/nu15051211

**Published:** 2023-02-28

**Authors:** Linzi Li, Pamela L. Lutsey, Lin Yee Chen, Elsayed Z. Soliman, Mary R. Rooney, Alvaro Alonso

**Affiliations:** 1 Department of Epidemiology, Rollins School of Public Health, Emory University, Atlanta, GA 30322, USA; 2 Division of Epidemiology and Community Health, School of Public Health, University of Minnesota Twin City, Minneapolis, MN 55455, USA; 3 Lillehei Heart Institute and Cardiovascular Division, Department of Medicine, University of Minnesota Medical School, Minneapolis, MN 55455, USA; 4 Epidemiological Cardiology Research Center, Section on Cardiovascular Medicine, Department of Medicine, Wake Forest School of Medicine, Winston-Salem, NC 27101, USA; 5 Department of Epidemiology, Johns Hopkins Bloomberg School of Public Health, Johns Hopkins University, Baltimore, MD 21205, USA

**Keywords:** circulating magnesium, atrial fibrillation, major adverse cardiac events, cardiovascular disease, observational prospective study

## Abstract

**Background:** Serum magnesium (Mg) has been reported to be inversely associated with the risk of atrial fibrillation (AF), coronary artery disease (CAD), and major adverse cardiovascular events (MACE). The association between serum Mg and the risk of MACE, heart failure (HF), stroke, and all-cause mortality among patients with AF has not been evaluated. **Objective:** We aim to examine whether higher serum Mg is associated with a lower risk of MACE, heart failure (HF), stroke, and all-cause mortality among patients with AF. **Methods:** We evaluated prospectively 413 participants of the Atherosclerosis Risk in Communities (ARIC) Study with a diagnosis of AF at the time of Mg measurement participating in visit 5 (2011–2013). Serum Mg was modeled in tertiles and as a continuous variable in standard deviation units. Endpoints (HF, MI, stroke, cardiovascular (CV) death, all-cause mortality, and MACE) were identified and modeled separately using Cox proportional hazard regression adjusting for potential confounders. **Results:** During a mean follow-up of 5.8 years, there were 79 HFs, 34 MIs, 24 strokes, 80 CV deaths, 110 MACEs, and 198 total deaths. After adjustment for demographic and clinical variables, participants in the second and third tertiles of serum Mg had lower rates of most endpoints, with the strongest inverse association for the incidence of MI (HR 0.20, 95% CI 0.07, 0.61) comparing top to bottom tertile. Serum Mg modeled linearly as a continuous variable did not show clear associations with endpoints except MI (HR 0.50, 95% CI 0.31, 0.80). Due to the limited number of events, the precision of most estimates of association was relatively low. **Conclusions:** Among patients with AF, higher serum Mg was associated with a lower risk of developing incident MI and, to a lesser extent, other CV endpoints. Further studies in larger patients with AF cohorts are needed to evaluate the role of serum Mg in preventing adverse CV outcomes in these patients.

## 1. Background

Magnesium (Mg), as one of the most abundant cations in the human body, plays a critical role in several physiological, biochemical, and cellular processes that regulate cardiovascular function. Mg is related to the pathogenesis and mechanism of some cardiovascular diseases, such as heart failure (HF), hypertension, and cardiac arrhythmias [1]. Mg exerts antiarrhythmic effects on cells through modulation of myocardial excitability, influencing the risk of cardiac arrhythmias [2]. Existing literature has documented that serum Mg deficiency could identify patients with a higher risk of postoperative atrial fibrillation (AF) after coronary artery bypass surgery [3,4]. Some studies also suggest that Mg supplementation reduces AF incidence after cardiac surgery [5,6,7]. In the community-based Framingham Offspring Heart Study and the Atherosclerosis Risk in Communities (ARIC) Study, an inverse association between serum Mg and the risk of AF has been described [8,9,10].

Serum Mg is also related to other cardiovascular conditions [11]. Evidence shows that serum Mg was associated with coronary heart and vascular disease deaths and hospitalizations and all-cause mortality inversely in a nationally representative population-based sample [12,13]. Low serum Mg also independently predicted all-cause and cardiovascular mortality, adjusting for established cardiovascular risk factors [14]. Among patients with myocardial infarction (MI), low serum Mg was associated with major adverse cardiac events (MACE), including death, recurrent MI, stroke, and any revascularization [15].

AF shares risk factors and usually co-exists with coronary artery disease (CAD) [16]. However, there is little information regarding the association between serum Mg and MACE among patients with AF. Using data from the ARIC study, which has obtained information on cardiovascular risk factors, AF, and other cardiac events endpoints, we investigated how serum Mg is related to MACE, heart failure (HF), and all-cause mortality among patients with AF. We hypothesized that lower serum Mg concentrations are associated with a higher risk of MACE, HF, and all-cause mortality among patients with AF.

## 2. Methods

### 2.1. Study Population and Design

**Figure 1:**
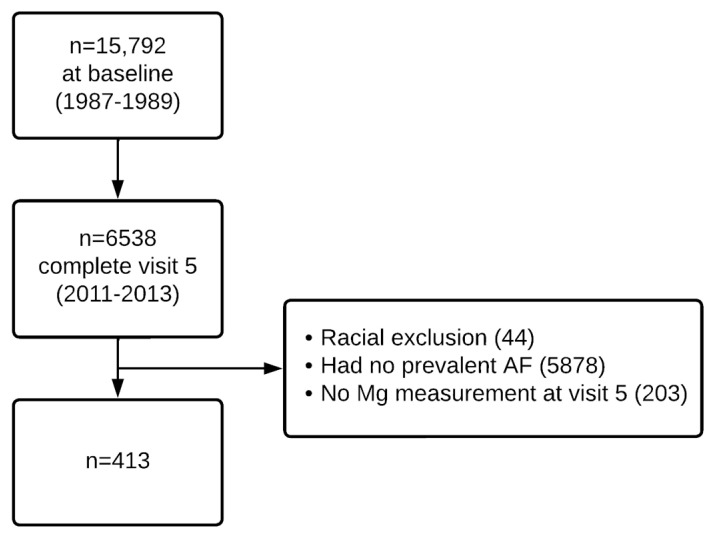
Flowchart of study participants inclusion, ARIC study. Racial exclusion: non-whites from the Minneapolis and Washington County field centers, and individuals who self-reported their race as other than white or African American in the Forsyth County field center.

The ARIC Study is a community-based prospective cohort conducted in four United States communities: Forsyth County, North Carolina; Washington County, Maryland; selected Minneapolis suburbs, Minnesota; and Jackson, Mississippi. The study aims to investigate cardiovascular risk factors. The detailed study design was described elsewhere [17]. In 1987–1989, approximately 4000 individuals aged 45–64 years in each field center were enrolled. In total, 15,792 participants completed an extensive baseline examination. After the baseline examination (visit 1), participants attended follow-up visits, occurring in 1990–1992 (visit 2), 1993–1995 (visit 3), 1996–1998 (visit 4), 2011–2013 (visit 5), 2016–2017 (visit 6), and 2018–2019 (visit 7). During each study visit, information on clinical and lifestyle variables was collected. In this study, we included those with available serum Mg measurements and diagnosis of AF at the time of examination in visit 5. At other visits, no Mg concentration was measured or measured using the same assay. We excluded those who had missing serum Mg concentrations, those who did not have AF or had missing AF status at the time Mg was measured, as well as non-whites from the Minneapolis and Washington County field centers, and individuals who self-reported their race as other than white or African American in the Forsyth County field center because of small numbers in some race-center combinations. This resulted in 413 unique individuals in the analytic dataset (Figure 1). All the participants were followed until the end of 2019 (end of 2017 for Jackson participants). Institutional review boards approved the study protocol at participating institutions. All study participants provided written informed consent.

### 2.2. Assessment of Serum Mg Concentrations

At visit 5, participants had blood samples collected after eight hours of fasting following standardized protocols. Serum samples were stored at −80 °C and shipped to a central facility. In 2016, Mg concentrations were measured using these serum samples with a colorimetric (xylidyl blue) method in Roche COBAS 6000 chemistry analyzer (Roche Diagnostics, Indianapolis, IN, USA). The coefficient of variation was 1.9% using measurements from 242 duplicates [18]. Circulating Mg refers to the total blood level in this study.

### 2.3. Definition of Prevalent AF and Incident Endpoints

Prevalent AF at visit 5 was defined as (1) electrocardiographic evidence of the arrhythmia at the current or prior study visits, or (2) between visits 1 and 5, any presence of AF in hospitalization discharge diagnosis not associated with open cardiac surgery [19].

The separate endpoints in this study were incident HF, MI, stroke, cardiovascular (CV) death, and all-cause mortality during follow-up. The combined endpoint was MACE (MI, stroke, cardiovascular death). Incident HF was determined as the first HF hospitalization or death due to HF. HF hospitalizations were identified if ICD-9-CM 428.xx or ICD-10-CM I50.xx codes were included as discharge diagnoses, and HF deaths were identified if ICD-9 428 or ICD-10 I50 codes were listed in the death certificate [20]. Incident MIs were defined as definite or probable MI, death due to coronary heart disease, or any electrocardiographic evidence of a silent MI detected [21]. Incident strokes were defined as definite or probable strokes by criteria used in the National Survey of Stroke [22]. CV deaths were defined as ICD-9 401-459 or ICD-10 I10-I99. Stroke and CV death cases were confirmed by a computerized algorithm and physician reviewer independently [21,23]. All death events were determined from death certificates where causes of death were coded using ICD-9 or ICD-10 [24].

### 2.4. Ascertainment of Other Covariates

The following variables measured at visit 5 were covariates in this study: age, sex, race, study center, body mass index (BMI), smoking status (current, former, never), alcohol drinking status (current, former, never), systolic blood pressure (SBP), diastolic blood pressure (DBP), low-density lipoprotein cholesterol (LDLc), high-density lipoprotein cholesterol (HDLc), diabetes history, use of anti-hypertension medication other than diuretics, use of diuretics (loop diuretics and other diuretics), use of blood lipid-lowering medication, estimated glomerular filtration rate (eGFR), serum potassium, serum creatinine, use of anticoagulants, use of aspirin, use of beta-blocker, use of angiotensin-converting enzyme (ACE) inhibitor, use of angiotensin II receptor antagonists, use of aldosterone antagonist, use of antiarrhythmics, MI history, stroke history, and HF history. All demographic variables were self-reported and clinical variables were measured at the visit examination. Diabetes was defined as fasting blood glucose ≥126 mg/dL, non-fasting blood glucose ≥200 mg/dL, use of glucose-lowering medication, or a self-reported physician diagnosis of diabetes. Participants were asked to bring any medications and supplements taken during the two weeks prior to the exam. Medication use was determined by staff review at the time of the visit.

### 2.5. Statistical Analysis

Serum Mg concentration was categorized based on approximate tertiles (1.2–1.9, 2.0, 2.1–2.7 mg/dL) and, separately, using the thresholds defining normal range (1.7 and 2.2 mg/dL) [25]. According to these categories, baseline characteristics of the study population were described. The follow-up time was days from visit 5 to any incident outcome, loss to follow-up, or 31 December 2019, whichever happened earlier. For each outcome-specific analysis (HF, stroke, MI), we excluded those who had a prior history before visit 5, but not for the combined endpoint (MACE). The incidence rates of each outcome were calculated. Cox proportional hazard regression was used to estimate the hazard ratios (HRs) and 95% confidence intervals (CIs) between serum Mg concentration and the incident endpoints. Serum Mg concentrations were modeled as tertiles, normal range categories, and continuously (per 1 SD or ~0.21 mg/dL). The following models were run for each outcome: (1) crude model, (2) adjusted for age, sex, race, and study center, (3) model 2 plus adjustment for BMI, smoking status, drinking status, SBP, DBP, LDL, HDL, diabetes, use of diuretics and antihypertensive medication (loop diuretics, other diuretics, other antihypertensive medication, and no antihypertensive medication), use of lipid-lowering medications, eGFR, serum potassium, serum creatinine, use of anticoagulants and use of aspirin, and (4) model 3 plus an adjustment for MI history, stroke history, and HF history. Additionally, we used restricted cubic spline functions with 3 knots to visually examine the associations between serum Mg concentration and the incident endpoints. Direct adjusted survival curves were plotted for the incident endpoints using a SAS macro [26]. We also ran an additional model for each endpoint, adjusting for covariates in model 3 and beta-blocker use, ACE inhibitor use, angiotensin II receptor antagonist use, aldosterone antagonist use, and antiarrhythmic agent use. All the analyses were completed with SAS statistical software (v. 9.4, SAS Institute Inc., Cary, NC, USA).

### 2.6. Results

Characteristics of 413 ARIC participants grouped by Mg concentration tertiles and categories are shown in Table 1 and Supplemental Table S1. The tertile ranges were 1.2–1.9, 2.0, and 2.1–2.7 mg/dL. The number of participants in tertiles was unequal because tied Mg values were assigned to the same group. Those with normal serum Mg levels (1.7–2.2 mg/dL) were more likely to be male, and those with lower serum Mg levels (<1.7 mg/dL) were more likely to be younger, white, and have higher BMI.

Over an average follow-up of 5.7 years, 79 HFs, 34 MIs, 24 strokes, 80 CV deaths, 110 MACEs, and 198 total deaths were identified in the study population (Table 2). There tended to be a linear dose-response inverse association of serum Mg tertiles with incident MI, U-shape associations with incident stroke, CV death, MACE, and all-cause mortality, and an L-shape association with HF (Supplemental Figure S1). In the models adjusted for age, sex, race, and study center, participants in the second tertile of serum Mg had a lower risk of MI, MACE, and all-cause mortality than patients in the first tertile. The HRs (95% CIs) for MI, MACE, and all-cause mortality were 0.36 (0.14, 0.95), 0.48 (0.28, 0.83), and 0.62 (0.42, 0.92), respectively. There was an inverse association comparing participants in the third tertile and the first tertile for incident MI in all models adjusted for covariates [HRs (95% CI): model 1 0.43 (0.10, 0.93), model 2 0.23 (0.08, 0.65), model 3 0.20 (0.07, 0.61)]. There was evidence of dose-response for incident MI in model 2 and model 3. However, in other models, associations for individuals in the second and third tertiles of Mg level were attenuated and did not show statistically significant differences in the risks of HF, MI, stroke, CV death, MACE, and all-cause mortality. As 1 SD (~0.21 mg/dL) increased in Mg concentration, the risk of MI decreased by 50% (HR 0.50, 95% CI 0.31, 0.80). Mg concentration modeled as a continuous variable was not associated with the risk of incident HF, stroke, and MACE, nor with CV death and all-cause mortality.

In a secondary analysis using normal serum Mg level (1.7–2.2 mg/dL) as the reference category, there were no statistically significant differences in the risk of HF, MI, stroke, MACE, CV death, or all-cause mortality among individuals with low (<1.7 mg/dL) or high (>2.2 mg/dL) serum Mg (Supplemental Table S2). Adjusting for additional medication use did not change the results significantly (Supplemental Table S3).

## 3. Discussion

Our study found that higher serum Mg was associated with a lower risk of most cardiovascular outcomes (MI, HF, MACE, CV death, and all-cause mortality) in patients with AF, but showed a consistently significant association with incident MI after adjusting for other cardiovascular risk factors which may mediate the association between Mg and incident cardiovascular outcomes.

To our knowledge, literature documenting the association between circulating Mg concentrations and cardiac events among patients with AF in the long term is exiguous. Previous studies in ARIC have reported that low serum Mg was associated with an increased risk of incident HF [27], sudden cardiac death [28], and CHD, including definite or probable MI or definite CHD death [29]. In other studies, an inverse association between serum Mg and CV death and all-cause mortality has been reported [13,14]. We observed an inverse association between Mg and most cardiovascular outcomes in demographic-adjusted models, but not after adjusting for additional CVD risk factors which may mediate the association between Mg and CVD. The lack of independent significant associations for most outcomes in our study could be due to several reasons. First, the association of serum Mg among patients with AF with CV endpoints could be different from that in a healthy population. Second, the study population was relatively old, with a high prevalence of multiple chronic health conditions. The effect of other cardiovascular risk factors could overwhelm potential causal pathways, reducing the absolute impact of serum Mg. Third, the limited number of events reduced the precision of effect estimates. Some of the outcomes’ associations were in the hypothesized direction, though not statistically significant.

Limited evidence suggests that in the acute management of non-postoperative acute AF, Mg therapy does not perform better than placebo in preventing major adverse outcomes, including death (RR 0.85, 95% 0.44–1.61 in a metanalysis of 273 patients and 29 events) [30]. However, due to the small number of patients and events in this meta-analysis, the lack of a statistically significant association does not imply evidence of no effect of Mg on CV outcomes among patients with AF. Thus, further investigation with larger sample sizes is warranted in order to evaluate the role of serum Mg and Mg supplementation among those with existing AF as a secondary prevention strategy.

Previous epidemiological studies have demonstrated an association between serum Mg and hypertension in different populations, implying that Mg may play a role in regulating blood pressure through vascular smooth muscle cell relaxation [31,32,33]. Evidence showed that intracellular and extracellular Mg deficiency might participate in insulin resistance and metabolic syndrome development [34,35,36]. Improved low-grade inflammation was suggested as the potential mechanism by which Mg had a beneficial effect on hypertension, type 2 diabetes, and metabolic syndrome [35]. However, it remains unclear how serum Mg influences the pathophysiology of incident MI, especially among AF patients. Since hypertension, type 2 diabetes, and metabolic syndrome are common complications of MI, Mg might affect incident MI development by improving systemic inflammation and endothelial function.

There were strengths in our study, including the use of a community-based study sample, assessment of AF and cardiac events endpoint, measurements of cardiovascular risk factors allowing control for confounding, and the long follow-up. Our study also had limitations. First, the age of the study population was 78 years on average, limiting the generalizability of the results to populations in other age ranges. Second, for incident stroke and MI, the number of events was relatively small, resulting in imprecise confidence intervals. Third, due to the observational study design, the causal inference between serum Mg and cardiovascular outcomes is limited.

In conclusion, among patients with existing AF, higher serum Mg was associated with a lower risk of incident MI, but no significant association was observed with stroke, MACE, HF, and all-cause mortality. To better understand whether serum Mg and Mg therapy has a role in the prevention of MACE in patients with AF, further studies in larger patient samples are needed.

The authors’ responsibilities were as follows: LL and AA designed the study; LL conducted the literature search, performed the statistical analysis, drafted the paper, and had primary responsibility for the final content; and all authors wrote the paper and read and approved the final manuscript. None of the authors reported a conflict of interest related to the study.

## Figures and Tables

**Table 1:** Visit 5 characteristics of participants in ARIC grouped by Mg concentration tertiles (*n* = 413).

	Mg Level Tertiles (mg/dL)		
	T1	T2	T3
	(1.2–1.9)	2.0	(2.1–2.7)
*n*	157	98	158
Age, years	77.6 (5.3)	78.4 (5.5)	78.4 (5.2)
Sex, % women	83 (52.9)	39 (39.8)	65 (41.1)
Race, % African American	22 (14)	16 (16.3)	20 (12.7)
Body mass index, kg/m^2^	30.4 (7.1)	29.1 (5.7)	28.2 (5.1)
Systolic blood pressure, mmHg	128.9 (20.5)	128.1 (18)	127.1 (18.5)
High-density lipoprotein, mg/L	48.2 (12.8)	50 (14.7)	50.3 (14.3)
Low-density lipoprotein, mg/L	86.5 (29.3)	94.7 (29)	97.8 (38.3)
eGFR, mL/min per 1.73 m^2^	59.2 (16.1)	58.7 (19)	55.1 (18.3)
Smoking status			
Current smoker	9 (6.3)	5 (5.4)	8 (5.8)
Former smoker	81 (57)	53 (57)	87 (63)
Never smoker	52 (36.6)	35 (37.6)	43 (31.2)
Drinking status			
Current drinker	76 (50.7)	47 (49.5)	67 (45)
Former drinker	48 (32)	23 (24.2)	53 (35.6)
Never drinker	26 (17.3)	25 (26.3)	29 (19.5)
Blood cholesterol treatment, %	109 (69.9)	57 (59.4)	97 (61.8)
Use of diuretics, %	66 (42)	38 (38.8)	63 (39.9)
Hypertension treatment, %	148 (94.3)	85 (86.7)	138 (87.3)
Serum potassium, mg/L	4 (0.4)	4.1 (0.4)	4.1 (0.4)
Serum creatinine, mg/L	1 (0.3)	1.1 (0.3)	1.2 (0.6)
Aspirin use, %	102 (65.4)	63 (65.6)	113 (72)
Anticoagulant use, %	86 (55.1)	55 (57.3)	75 (47.8)
Antiarrhythmics use, %	1 (0.6)	3 (3.1)	1 (0.6)
Beta-blocker use, %	102 (65.4)	50 (52.1)	98 (62.4)
Angiotensin-converting enzyme inhibitor use, %	65 (41.7)	36 (37.5)	48 (30.6)
Angiotensin II receptor antagonists use, %	23 (14.7)	11 (11.5)	20 (12.7)
Aldosterone antagonist use, %	12 (7.7)	5 (5.2)	7 (4.5)
Diabetes, %	93 (60.8)	29 (30.5)	42 (27.1)
MI history, %	7 (4.8)	2 (2.2)	4 (2.7)
Stroke history, %	13 (8.3)	14 (14.3)	8 (5.1)
HF history, %	36 (22.9)	22 (22.5)	42 (26.6)

Notes: Data are shown as frequency (percentage) or mean (SD).

**Table 2:** Association between serum Mg and major adverse cardiovascular events among participants with prevalent atrial fibrillation, ARIC (*n* = 413).

		Mg Level Tertiles (mg/dL)			Continuous
		T1	T2	T3	per SD (0.2078)
		(1.2–1.9)	2	(2.1–2.7)	
HF (*n* = 308)					
	Cases, *n*	31	21	27	79
	Incidence rate, /1000 pys	53.78	51.29	40.05	47.59
	Crude model	Ref	0.93 (0.54, 1.62)	0.72 (0.43, 1.20)	0.85 (0.67, 1.07)
	Model 1	Ref	0.65 (0.36, 1.16)	0.59 (0.34, 1.00)	0.80 (0.63, 1.01)
	Model 2	Ref	0.56 (0.29, 1.06)	0.62 (0.34, 1.15)	0.85 (0.64, 1.12)
	Model 3	Ref	0.60 (0.31, 1.16)	0.65 (0.34, 1.22)	0.85 (0.64, 1.14)
MI (*n* = 346)					
	Cases, *n*	17	6	11	34
	Incidence rate, /1000 pys	25.32	12.70	13.36	17.28
	Crude model	Ref	0.48 (0.19, 1.23)	0.50 (0.23, 1.07)	0.84 (0.60, 1.18)
	Model 1	Ref	0.36 (0.14, 0.95)	0.43 (0.20, 0.93)	0.80 (0.57, 1.13)
	Model 2	Ref	0.34 (0.12, 1.00)	0.23 (0.08, 0.65)	0.54 (0.35, 0.84)
	Model 3	Ref	0.36 (0.12, 1.07)	0.20 (0.07, 0.61)	0.50 (0.31, 0.80)
Stroke (*n* = 378)					
	Cases, *n*	9	4	11	24
	Incidence rate, /1000 pys	11.69	7.99	12.84	11.28
	Crude model	Ref	0.68 (0.21, 2.22)	0.84 (0.60, 1.18)	1.15 (0.74, 1.76)
	Model 1	Ref	0.50 (0.15, 1.68)	0.90 (0.37, 2.21)	1.06 (0.68, 1.65)
	Model 2	Ref	0.65 (0.17, 2.50)	0.97 (0.32, 2.95)	1.09 (0.63, 1.87)
	Model 3	Ref	0.71 (0.17, 2.92)	0.94 (0.31, 2.9)	1.07 (0.60, 1.89)
Cardiovascular death (*n* = 413)					
	Cases, *n*	29	13	38	80
	Incidence rate, /1000 pys	33.18	22.39	41.01	33.60
	Crude model	Ref	0.66 (0.35, 1.28)	1.21 (0.75, 1.97)	1.24 (0.97, 1.57)
	Model 1	Ref	0.55 (0.28, 1.07)	1.03 (0.63, 1.68)	1.14 (0.90, 1.45)
	Model 2	Ref	0.73 (0.35, 1.54)	0.99 (0.52, 1.89)	1.13 (0.82, 1.56)
	Model 3	Ref	0.69 (0.31, 1.51)	1.01 (0.52, 1.96)	1.15 (0.82, 1.62)
MACE (*n* = 413)					
	Cases, *n*	45	19	46	110
	Incidence rate, /1000 pys	53.12	33.22	51.30	47.50
	Crude model	Ref	0.61 (0.36, 1.04)	0.95 (0.63, 1.43)	1.12 (0.91, 1.37)
	Model 1	Ref	0.48 (0.28, 0.83)	0.80 (0.52, 1.22)	1.04 (0.85, 1.27)
	Model 2	Ref	0.68 (0.37, 1.26)	0.81 (0.48, 1.39)	1.02 (0.79, 1.33)
	Model 3	Ref	0.66 (0.35, 1.24)	0.79 (0.46, 1.36)	1.01 (0.77, 1.32)
All-cause mortality (*n* = 413)					
	Cases, *n*	80	39	79	198
	Incidence rate, /1000 pys	90.58	67.16	85.03	82.74
	Crude model	Ref	0.73 (0.50, 1.07)	0.92 (0.68, 1.26)	0.95 (0.83, 1.10)
	Model 1	Ref	0.62 (0.42, 0.92)	0.79 (0.58, 1.09)	0.90 (0.78, 1.04)
	Model 2	Ref	0.76 (0.49, 1.19)	0.87 (0.59, 1.30)	0.89 (0.73, 1.07)
	Model 3	Ref	0.78 (0.49, 1.24)	0.91 (0.61, 1.37)	0.90 (0.74, 1.09)

Notes: Results are shown as HR (95% CI). Model 1: crude model + sex, race, age, study center. Model 2: model 1 + BMI, drinking status, smoking status, SBP, DBP, LDL, HDL, diabetes, eGFR, use of blood cholesterol medications, serum potassium, serum creatinine, use of anticoagulants, use of aspirin, use of diuretics and antihypertensive medication (loop diuretics, other diuretics, other antihypertensive medication, and no antihypertensive medication). Model 3: Model 2 + MI history, stroke history, HF history. For each outcome-specific analysis (HF, stroke, MI) we excluded those who had a prior history before visit 5, but not for the combined endpoint.

## Data Availability

Data sharing can be conducted through the ARIC Coordinating Center: https://sites.cscc.unc.edu/aric/distribution-agreements.

## Supplementary Materials

The following supporting information can be downloaded at: https://www.mdpi.com/article/10.3390/nu15051211/s1, Table S1: Visit 5 characteristics of participants in ARIC grouped by Mg concentration levels (N = 413); Table S2. Association between serum Mg and major adverse cardiovascular events among participants with prevalent atrial fibrillation, ARIC (N = 413); Figure S1 (**a**–**f**). Association of serum Mg concentration with incidence of (**a**) HF; (**b**) MI; (**c**) stroke; (**d**) CV death; (**e**) MACE; (**f**) all-cause mortality presented as ln(HR) (red line) and 95% confidence intervals (dotted line). Figure S2 (**a**–**f**). Direct adjusted survival curves for (**a**) HF; (**b**) MI; (**c**) stroke; (**d**) CV death; (**e**) MACE; (**f**); Table S3. Association between serum Mg and major adverse cardiovascular events among participants with prevalent atrial fibrillation, adjusting for additional medications, ARIC (N = 413).

## Abbreviations

Mg: magnesium; ARIC, Atherosclerosis Risk in Communities; AF, atrial fibrillation; MACE, major adverse cardiac events; CAD, coronary artery disease; CVD, cardiovascular disease; HF, heart failure; MI, myocardial infarction; eGFR, estimated glomerular filtration rate; MI, myocardial infarction.
