# Supplementary material for: Circulating Magnesium and Risk of Major Adverse Cardiac Events among Patients with Atrial Fibrillation in the ARIC Cohort
Source: Nutrients. 2023 Feb 28;15(5):1211. doi: 10.3390/nu15051211 (PMC10005106; doi:10.3390/nu15051211)
Supplement: Supplementary file 1 [file nutrients-15-01211-s001.zip › nutrients-2170746-supplementary.pdf]

Supplemental Table S1. Visit 5 characteristics of participants in ARIC grouped by Mg concentration levels (N=413)

	Mg concentration level (mg/dL)		
	< 1.7	1.7-2.2	> 2.2
N	27	353	33
Age, years	77.5 (4.4)	77.9 (5.4)	80.2 (5.2)
Sex, %women	14 (51.9)	154 (43.6)	19 (57.6)
Race, %African American	2 (7.4)	47 (13.3)	9 (27.3)
Body mass index, kg/m^2	30 (7.3)	29.3 (6.1)	27.9 (6)
Systolic blood pressure, mmHg	131 (27.7)	127.4 (18)	132.4 (22.7)
High density lipoprotein, mg/L	45.4 (13.4)	49.2 (13.7)	54.9 (14.5)
Low density lipoprotein, mg/L	77.3 (27.4)	93.2 (32.6)	100.3 (41.6)
eGFR, mL/min per 1.73 m^2	63.1 (16.8)	58.7 (16.8)	40.7 (19.9)
Smoking Status			
Current smoker	2 (8)	19 (6)	1 (3.3)
Former smoker	15 (60)	188 (59.1)	18 (60)
Never smoker	8 (32)	111 (34.9)	11 (36.7)
Drinking Status			
Current drinker	13 (50)	166 (49.3)	11 (35.5)
Former drinker	10 (38.5)	102 (30.3)	12 (38.7)
Never drinker	3 (11.5)	69 (20.5)	8 (25.8)
Blood cholesterol treatment, %	22 (81.5)	221 (63.3)	20 (60.6)
Use of diuretics, %	5 (18.5)	149 (42.2)	13 (39.4)
Hypertension treatment, %	25 (92.6)	316 (89.5)	30 (90.9)
Serum potassium, mg/L	4 (0.3)	4.1 (0.4)	4.1 (0.4)
Serum creatinine, mg/L	1 (0.3)	1.1 (0.3)	1.7 (1)
Aspirin use, %	18 (66.7)	234 (67.1)	26 (78.8)

Anticoagulant use, %	16 (59.3)	186 (53.3)	14 (42.4)
Antiarrhythmics use, %	0	4 (1.1)	1 (3.0)
Beta-blocker use, %	18 (66.7)	213 (61)	19 (57.6)
Angiotensin converting enzyme inhibitor use, %	10 (37)	129 (37)	10 (30.3)
Angiotensin II receptor antagonists use, %	4 (14.8)	46 (13.2)	4 (12.1)
Aldosterone antagonist use, %	1 (3.7)	22 (6.3)	1 (3)
Diabetes, %	18 (72)	135 (39)	11 (34.4)
MI history, %	0	13 (4)	0
Stroke history, %	5 (18.5)	28 (7.9)	2 (6.1)
HF history, %	7 (25.9)	81 (23)	12 (36.4)

Notes: Data are shown as frequency (percentage) or mean (SD).

Supplemental Table S2. Association between serum Mg and major adverse cardiovascular events among participants with prevalent atrial fibrillation, ARIC (N=413)

		Mg concentration level (mg/dL)		
		< 1.7	1.7-2.2	> 2.2
HF (N=308)				
	Cases, n	6	68	5
	Incidence rate, /1000 pys	88.67	45.90	45.02
	Crude model	2.06 (0.89, 4.77)	Ref	0.98 (0.40, 2.44)
	Model 1	2.16 (0.92, 5.07)	Ref	1.04 (0.42, 2.58)
	Model 2	2.30 (0.84, 6.27)	Ref	0.76 (0.22, 2.71)
	Model 3	2.41 (0.87, 6.70)	Ref	0.83 (0.23, 2.96)
MI (N=346)				
	Cases, n	3	27	4

	Incidence rate, /1000 pys	27.40	15.73	28.32
	Crude model	1.87 (0.57, 6.20)	Ref	1.82 (0.64, 5.21)
	Model 1	1.97 (0.57, 6.79)	Ref	1.93 (0.67, 5.59)
	Model 2	4.63 (1.06, 20.23)	Ref	1.18 (0.29, 4.79)
	Model 3	4.49 (1.01, 20.01)	Ref	1.01 (0.24, 4.30)
Stroke(N=378)				
	Cases, n	2	18	4
	Incidence rate, /1000 pys	17.41	9.70	25.72
	Crude model	1.85 (0.43, 7.99)	Ref	2.70 (0.91, 8.00)
	Model 1	2.20 (0.49, 9.93)	Ref	2.57 (0.84, 7.86)
	Model 2	3.87 (0.68, 22.13)	Ref	3.63 (0.81, 16.25)
	Model 3	5.44 (0.90, 32.76)	Ref	5.29 (1.12, 25.09)
Cardiovascular death (N=413)				
	Cases, n	3	63	14
	Incidence rate, /1000 pys	20.19	30.59	80.72
	Crude model	0.68 (0.21, 2.16)	Ref	2.69 (1.51, 4.81)
	Model 1	0.75 (0.23, 2.40)	Ref	2.28 (1.26, 4.12)
	Model 2	1.26 (0.36, 4.43)	Ref	3.15 (1.40, 7.08)
	Model 3	1.12 (0.31, 4.05)	Ref	2.99 (1.27, 7.02)
MACE (N=413)				
	Cases, n	5	88	17
	Incidence rate, /1000 pys	34.30	43.94	101.42
	Crude model	0.84 (0.34, 2.07)	Ref	2.42 (1.44, 4.08)
	Model 1	0.95 (0.38, 2.37)	Ref	2.07 (1.21, 3.52)
	Model 2	1.42 (0.53, 3.80)	Ref	2.73 (1.36, 5.49)
	Model 3	1.45 (0.53, 3.98)	Ref	2.54 (1.23, 5.27)
All-cause mortality(N=413)				

Cases, n	14	164	20
Incidence rate, /1000 pys	93.11	79.26	115.31
Crude model	1.21 (0.70, 2.08)	Ref	1.49 (0.94, 2.37)
Model 1	1.28 (0.74, 2.23)	Ref	1.31 (0.82, 2.10)
Model 2	1.74 (0.91, 3.33)	Ref	1.36 (0.71, 2.60)
Model 3	1.66 (0.85, 3.24)	Ref	1.28 (0.65, 2.51)

Notes: Results are shown as HR (95% CI)

model 1: crude model+sex, race, age, study center

model 2: model 1+ BMI, drinking status, smoking status, SBP, DBP, LDL, HDL, diabetes, eGFR, use of blood cholesterol medications, serum potassium, serum creatinine, use of anticoagulants, use of aspirin, use of diuretics and antihypertensive medication (loop diuretics, other diuretics, other antihypertensive medication, and no antihypertensive medication)

model 3: model 2 + MI history, stroke history, HF history

For each outcome-specific analysis (HF, stroke, MI) we excluded those who had prior history before visit 5, but not for the combined endpoint.

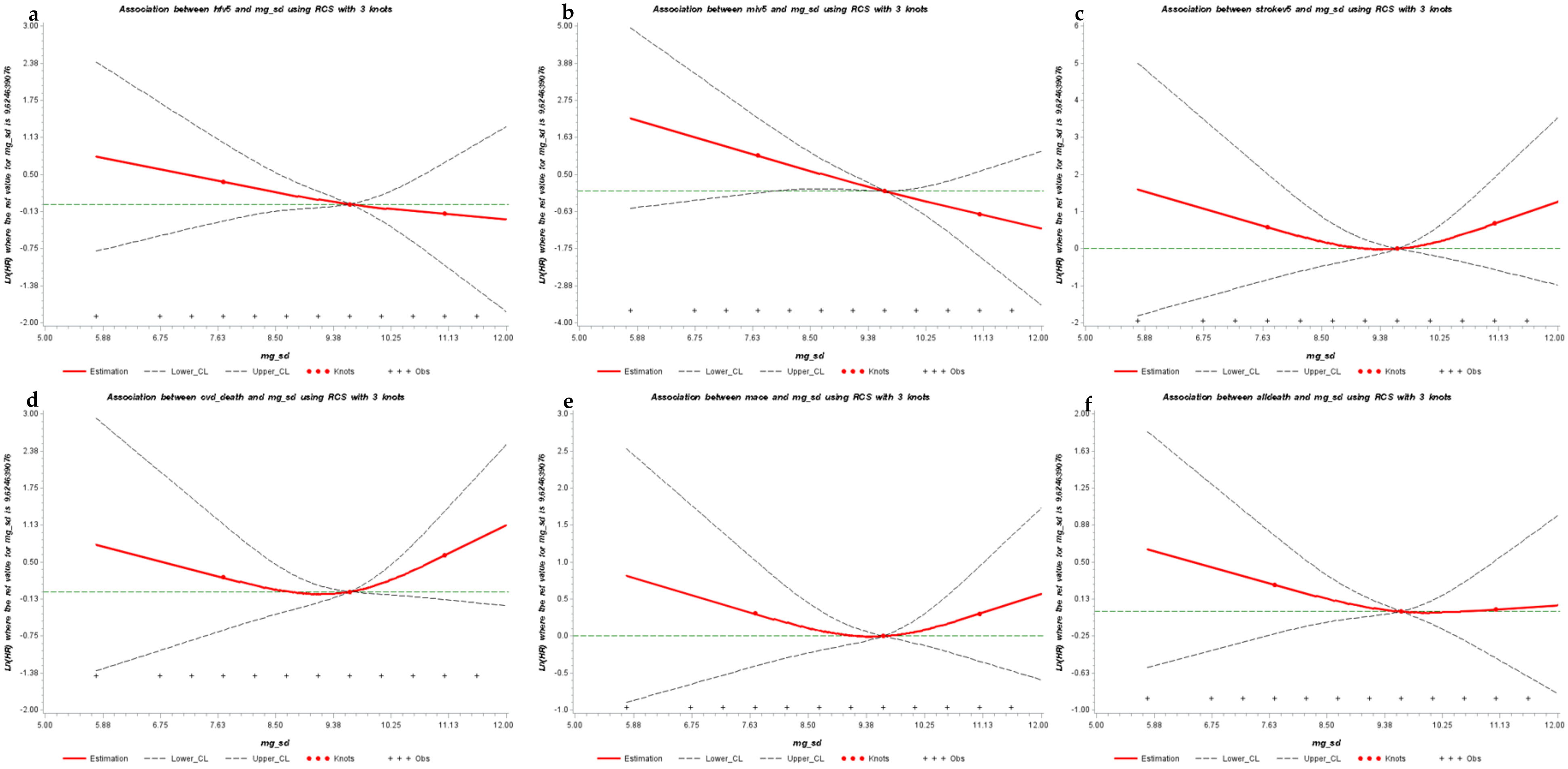

Supplemental Figure S1 (a-f). Association of serum Mg concentration with incidence of a) HF; b) MI; c) stroke; d) CV death; e) MACE; f) all-cause mortality presented as ln(HR) (red line) and 95% confidence intervals (dotted line). Results from Cox proportional hazards model with serum Mg concentration modeled using a

restricted cubic spline with 3 knots (25th, 50th, and 75th percentiles), adjusted for sex, race, age, study center, BMI, drinking status, smoking status, SBP, DBP, LDL, HDL, diabetes, eGFR, use of blood cholesterol medications, serum potassium, serum creatinine, use of anticoagulants, use of aspirin, use of diuretics and antihypertensive medication (loop diuretics, other diuretics, other antihypertensive medication, and no antihypertensive medication), MI history, stroke history, HF history

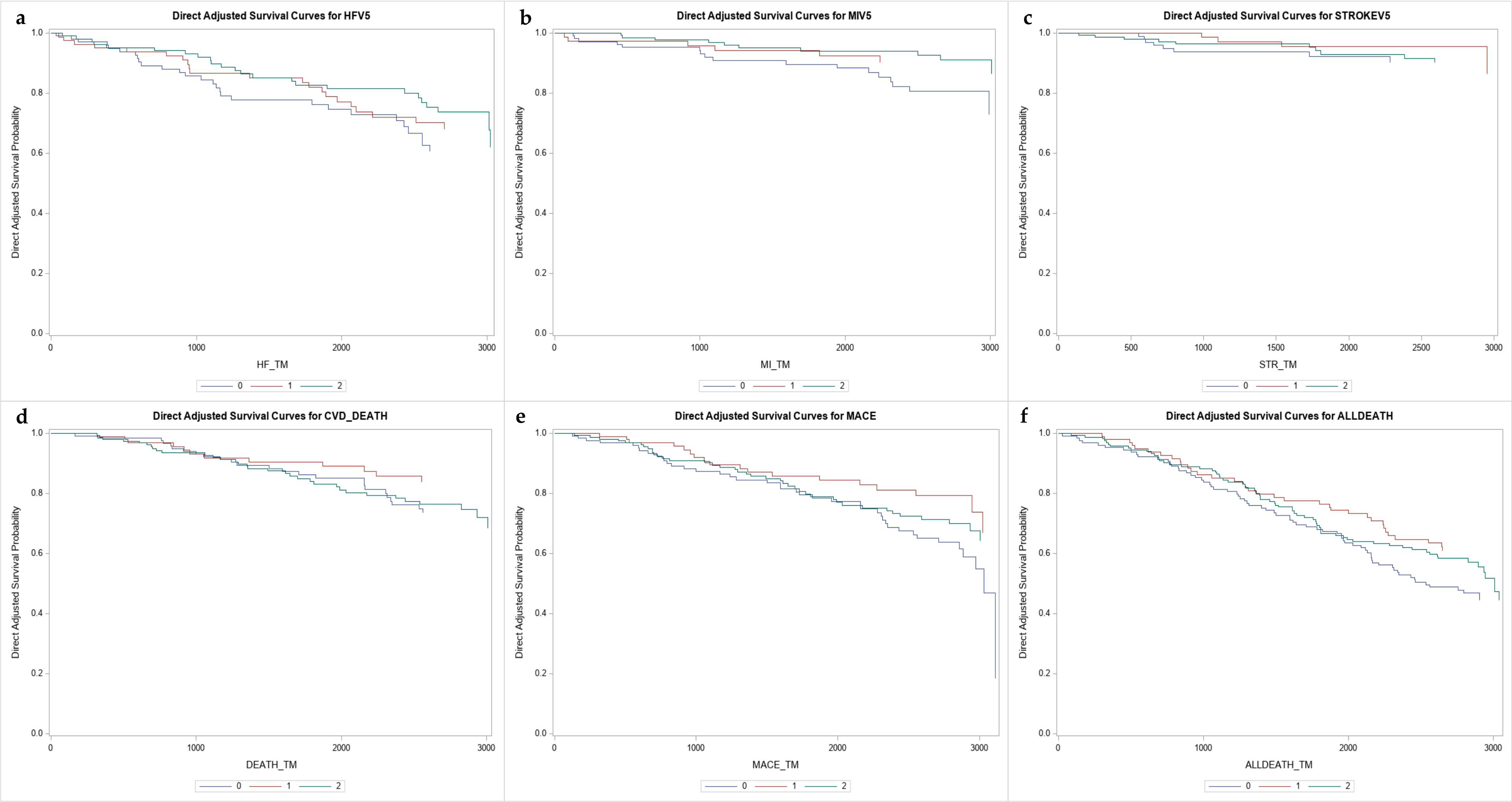
a
Direct Adjusted Survival Curves for HFV5
Direct Adjusted Survival Probability
1.0
0.8
0.6
0.4
0.2
0.0
0
1000
2000
3000
HF_TM
0
1
2
b
Direct Adjusted Survival Curves for MIV5
Direct Adjusted Survival Probability
1.0
0.8
0.6
0.4
0.2
0.0
0
1000
2000
3000
MI_TM
0
1
2
c
Direct Adjusted Survival Curves for STROKEV5
Direct Adjusted Survival Probability
1.0
0.8
0.6
0.4
0.2
0.0
0
500
1000
1500
2000
2500
3000
STR_TM
0
1
2
d
Direct Adjusted Survival Curves for CVD_DEATH
Direct Adjusted Survival Probability
1.0
0.8
0.6
0.4
0.2
0.0
0
1000
2000
3000
DEATH_TM
0
1
2
e
Direct Adjusted Survival Curves for MACE
Direct Adjusted Survival Probability
1.0
0.8
0.6
0.4
0.2
0.0
0
1000
2000
3000
MACE_TM
0
1
2
f
Direct Adjusted Survival Curves for ALLDEATH
Direct Adjusted Survival Probability
1.0
0.8
0.6
0.4
0.2
0.0
0
1000
2000
3000
ALLDEATH_TM
0
1
2

Supplemental Figure S2 (a-f). Direct adjusted survival curves for a) HF; b) MI; c) stroke; d) CV death; e) MACE; f) all-cause mortality, adjusted for sex, race, age, study center, BMI, drinking status, smoking status, SBP, DBP, LDL, HDL, diabetes, eGFR, use of blood cholesterol medications, serum potassium, serum creatinine, use of anticoagulants, use of aspirin, use of diuretics and antihypertensive medication (loop diuretics, other diuretics, other antihypertensive medication, and no antihypertensive medication), MI history, stroke history, HF history. 0,1, and 2 represent Mg concentration tertile.

Supplemental Table S3. Association between serum Mg and major adverse cardiovascular events among participants with prevalent atrial fibrillation, adjusting for additional medications, ARIC (N=413)

	Mg level tertiles (mg/dL)			Continuous
	T1 (1.2-1.9)	T2 2	T3 (2.1-2.7)	per SD (0.2078)
HF (N=308)				
	Ref	0.72 (0.36, 1.46)	0.70 (0.37, 1.33)	0.96 (0.71, 1.29)
MI (N=346)				
	Ref	0.34 (0.11, 1.05)	0.19 (0.06, 0.60)	0.52 (0.32, 0.84)
Stroke(N=378)				
	Ref	0.77 (0.18, 3.30)	1.15 (0.33, 3.98)	1.19 (0.65, 2.19)
Cardiovascular death (N=413)				
	Ref	0.82 (0.37, 1.86)	1.11 (0.56, 2.16)	1.20 (0.85, 1.68)
MACE (N=413)				
	Ref	0.77 (0.40, 1.47)	0.85 (0.49, 1.48)	1.04 (0.80, 1.36)
All-cause mortality(N=413)				
	Ref	0.79 (0.49, 1.26)	0.95 (0.63, 1.43)	0.92 (0.75, 1.11)

Notes: Results are shown as HR (95% CI)

Models were adjusted for sex, race, age, study center, BMI, drinking status, smoking status, SBP, DBP, LDL, HDL, diabetes,

eGFR, use of blood cholesterol medications, serum potassium, serum creatinine, use of anticoagulants, use of aspirin, use of diuretics and antihypertensive medication (loop diuretics, other diuretics, other antihypertensive medication, and no antihypertensive medication), MI history, stroke history, HF history, beta-blocker use, angiotensin converting enzyme inhibitor use, angiotensin II receptor antagonists use, aldosterone antagonist use, antiarrhythmics use.

For each outcome-specific analysis (HF, stroke, MI) we excluded those who had prior history before visit 5, but not for the combined endpoint.
